# Early Activation of FGF and Nodal Pathways Mediates Cardiac Specification Independently of Wnt/β-Catenin Signaling

**DOI:** 10.1371/journal.pone.0007650

**Published:** 2009-10-28

**Authors:** Lee J. Samuel, Branko V. Latinkić

**Affiliations:** School of Biosciences, Cardiff University, Cardiff, United Kingdom; The University of Hong Kong, China

## Abstract

**Background:**

Cardiac induction, the first step in heart development in vertebrate embryos, is thought to be initiated by anterior endoderm during gastrulation, but what the signals are and how they act is unknown. Several signaling pathways, including FGF, Nodal, BMP and Wnt have been implicated in cardiac specification, in both gain- and loss-of-function experiments. However, as these pathways regulate germ layer formation and patterning, their specific roles in cardiac induction have been difficult to define.

**Methodology/Principal Findings:**

To investigate the mechanisms of cardiac induction directly we devised an assay based on conjugates of anterior endoderm from early gastrula stage *Xenopus* embryos as the inducing tissue and pluripotent ectodermal explants as the responding tissue. We show that the anterior endoderm produces a specific signal, as skeletal muscle is not induced. Cardiac inducing signal needs up to two hours of interaction with the responding tissue to produce an effect. While we found that the BMP pathway was not necessary, our results demonstrate that the FGF and Nodal pathways are essential for cardiogenesis. They were required only during the first hour of cardiogenesis, while sustained activation of ERK was required for at least four hours. Our results also show that transient early activation of the Wnt/β-catenin pathway has no effect on cardiogenesis, while later activation of the pathway antagonizes cardiac differentiation.

**Conclusions/Significance:**

We have described an assay for investigating the mechanisms of cardiac induction by anterior endoderm. The assay was used to provide evidence for a direct, early and transient requirement of FGF and Nodal pathways. In addition, we demonstrate that Wnt/β-catenin pathway plays no direct role in vertebrate cardiac specification, but needs to be suppressed just prior to differentiation.

## Introduction

The earliest step in vertebrate heart formation, specification of cardiac progenitors, is poorly understood. We know that cardiac induction occurs during gastrulation, but the precise details of the timing and the nature of the signals are unknown. One reason for this low resolution picture is that the analyses of cardiac specification are necessarily retrospective, since there are no markers uniquely associated with cardiac fate that would allow cardiac progenitors to be traced from the time of their specification until they initiate terminal differentiation. Further complication arises from the fact that the signaling pathways implied in mediating cardiac specification frequently have multiple roles in the early embryo that are difficult to distinguish.

Numerous studies in the chick, mouse and *Xenopus* suggest cardiac tissue is induced by anterior endoderm (reviewed by [1]. Efficient cardiogenesis in *Xenopus* in vivo and in explants of the Dorsal Marginal Zone (DMZ) require anterior endoderm [2]. Similarly, endoderm is required for cardiac specification in chick embryos, and explants of early chick embryo hypoblast or anterior endoderm can promote cardiogenesis in non-cardiogenic mesoderm [3], [4]. In the mouse embryo, visceral endoderm, the tissue homologous to the chick anterior endoderm, is required for terminal differentiation of cardiomyocytes [5].

Proposed candidates for the cardiogenic inducing signal include Bone Morphogenetic Proteins (BMP's), Fibroblast Growth Factor (FGF) and Nodal/Activin. In the chick embryo, BMP's are necessary for cardiogenic activity of anterior endoderm in the chick embryo, while inhibitors of BMP activity suppress cardiac differentiation. [6], [7]. Zebrafish *swirl* (bmp2) mutant embryos have abnormal dorso-ventral patterning and lack cardiac progenitors marked by Nkx2.5 expression [8]. In *Xenopus* embryos, targeted interference of BMP signaling does not affect cardiac specification, but reduces differentiated cardiac muscle and disrupts heart morphogenesis [9], [10]. FGF signaling has also been implicated in cardiac specification in chick and zebrafish. A mutation in *acerebellar* (FGF8) gene in zebrafish embryos reduces the number of ventricular cardiomyocytes, and pharmacological inhibition of FGF signaling produces similar results [11], [12]. In chick embryos, ectopic FGF8 promotes lateral expansion of heart field, while ectopic FGF2 and 4, together with BMP4, promote cardiac differentiation in posterior non-cardiogenic mesoderm [13]–[15].

During early development of vertebrate embryos, specification of mesoderm and endoderm germ layers requires Nodal/Activin signalling pathway. As cardiac mesoderm is specified shortly afterwards, the precise role that the Nodal/Activin pathway plays in this process has been difficult to define. Nodal null mutant mouse embryos do not gastrulate, and hypomorphic mutants form abnormal hearts [16], [17]. Zebrafish embryos deficient for zygotic Nodal coreceptor *one-eyed pinhead (oep)* have reduced and abnormal hearts, along with other patterning defects, while the embryos completely devoid of *oep* (maternal and zygotic mutants) form no endoderm and dorsal mesoderm, including the heart [8], [18]. Evidence for a cardiac-specific role for Nodal/Activin signaling in cardiac specification comes from in vitro studies. Mouse ES cells deficient for Nodal coreceptor EGF-CFC (*Cripto*) are unable to form cardiomyocytes in vitro [19]. In addition, activin promotes cardiac differentiation of posterior epiblast explants of the chick [4] and induces cardiac tissue in a dose-dependent manner in *Xenopus* animal cap explants [20], [21]. While these studies suggest an instructive role of activin/Nodal pathway in cardiogenesis, questions of cell autonomy and specificity remain unanswered.

While BMP, FGF and the Nodal/Activin pathways are all implicated in promoting cardiac induction, there is evidence that the canonical Wnt signaling blocks and Wnt antagonism stimulates cardiogenesis in chick and *Xenopus* embryos and explants [22], [23]. In apparent contrast, Wnt/β-catenin signaling promotes cardiogenesis in ES cells if activated early, but opposes it when activated after specification [24], [25].

To avoid the difficulties associated with studying cardiac induction either in vivo or in complex explants, we have developed a simple assay for direct cardiac induction which uses anterior endoderm from early *Xenopus* gastrula as the inducer and pluripotent ectodermal explants as the responder.

## Materials and Methods

### *Xenopus* embryos and explants

All animal work was approved by Cardiff University's Ethical Review Committee and was undertaken under a licence from the UK Home Office.

Explants were dissected and cultured in 75% Normal Amphibian Media (NAM; [26] supplemented with gentamycin sulphate (50 µg/ml; Sigma). Anterior endoderm (AE) explants were dissected at stage 10–10.25 when the blastopore lip is discernible, using a fine tungsten needle and hair loop. Animal cap (AC) explants were routinely explanted at stage 8.5 [26]. Conjugation was achieved immediately upon dissection, with three animal caps per anterior endoderm. To age animal caps prior to conjugation they were cultured in Low Calcium-Magnesium Ringer's (LCMR; [26]) to prevent them from closing. Dissociation of the individual AE explants was carried out in Calcium-Magnesium free media (CMFM; [26]. Each AE was reaggregated in 75% NAM and subsequently conjugated to animal caps.

Inhibitors were dissolved in DMSO (except for LiCl, dissolved in water) and used at the following final concentration: SB-431542 (75 µM; Sigma [27]); A-83-01 (75 µM; Sigma [28]); SU5402 (50 µM; Calbiochem; [29]); U0126 (35 µM; Sigma [30]), 8 µM BIO (6-bromoindirubin-3′-oxime; [31] and 0.3 M LiCl [26], [32]. Treatments with LiCl and BIO were for 10 and 20 minutes, respectively. Control samples were incubated in DMSO.

### RNA and DNA injections

The templates used for mRNA synthesis were: Cerberus [33], Dkk-1 [34], Sox17βEnR [35], ΔFGFR1 [36], tBr [37], Cer-S [38], [39], Lef-β-catenin-GR [40], ΔActRI [41], constitutively active Alk4 receptor [42], ΔTcf3 [43]. RNA was subsequently purified on sephadex G50 columns (GE Healthcare). Unless otherwise stated, 1 ng of RNA was co-injected with a mix of rhodamine-dextran and dextran-biotin lineage tracers (Invitrogen) [26], [44]. CSKA-XWnt8 DNA [45] was used at 0.1 ng per embryo. Activity of constructs was verified by gene expression and phenotypic analysis.

### Gene Expression Analyses

#### RT-PCR

Total RNA was isolated from samples using the acid guanidinium thiocyanate-phenol-chloroform method [46]. 12–15 conjugates were used per sample, and cDNA synthesised using MMLV-RT (Invitrogen, UK) and random hexamers (Promega, UK) according to manufacturer's specifications. Primers were designed using Primer3 software (http://frodo.wi.mit.edu/cgi-bin/primer3/primer3_www.cgi) to span intron-exon boundaries of *X. tropicalis* orthologues, and PCR was carried out using GoTaq polymerase (Promega, UK) according to manufacturer instruction. Primer sequences and cycling conditions are described in the Table S1. Quantification of PCR products was achieved by gel densitometry using ImageJ software (http://rsbweb.nih.gov/ij/), normalizing against Ornithine Decarboxylase (ODC) expression. Linearity of gel RT-PCR and a comparison with quantitative RT-PCR are presented in Fig. S2. Quantitative RT-PCR was performed on a MiniOpticon Thermocycler (Bio-Rad) using SYBR Green kit (Bio-Rad). Samples were normalised to ODC and quantified using the Opticon Software (Bio-Rad). Samples were run in triplicate with the average reading shown.

Whole-mount *in situ* hybridization (WMISH) was carried out by the method described [26], using the digoxigenin (Roche)-labelled antisense riboprobe for cTnI [47] and MLC2 [48]. Colour reactions were performed using BM Purple (Roche). Following bleaching [26], injected biotinylated-dextran was revealed with ExtrAvidin-AP and FastRed substrate (Sigma and Roche, respectively). Conjugates were embedded in paraffin for sectioning, carried out by the Histology Unit, Cardiff School of Biosciences.

### Western Blotting

Western blotting was performed as described [49]. Total cell extracts were prepared from 10 µl lysis buffer/embryo, 1–2 µl lysis buffer/animal cap, or 6–8 µl lysis buffer/conjugate. 10 µl samples were resolved by polyacrylamide gel electrophoresis and transferred to PVDF membrane (Millipore) and blotted by standard techniques. Antibodies used were rat monoclonal α-HA-HRP (1∶2,500; Roche) and polyclonal α-diphosphorylated-ERK (1∶1,000; Cell-Signalling Technology). As a loading control, membranes were stripped and probed with α-Erk (1∶2,500; Santa-Cruz). Secondary antibody was goat α-rabbit HRP (Chemicon; 1∶5,000). Detection was achieved using chemiluminescent detection according to manufacturer (Pierce).

## Results

### Anterior endoderm is sufficient to induce cardiogenesis in animal cap cells

**Figure 1:**
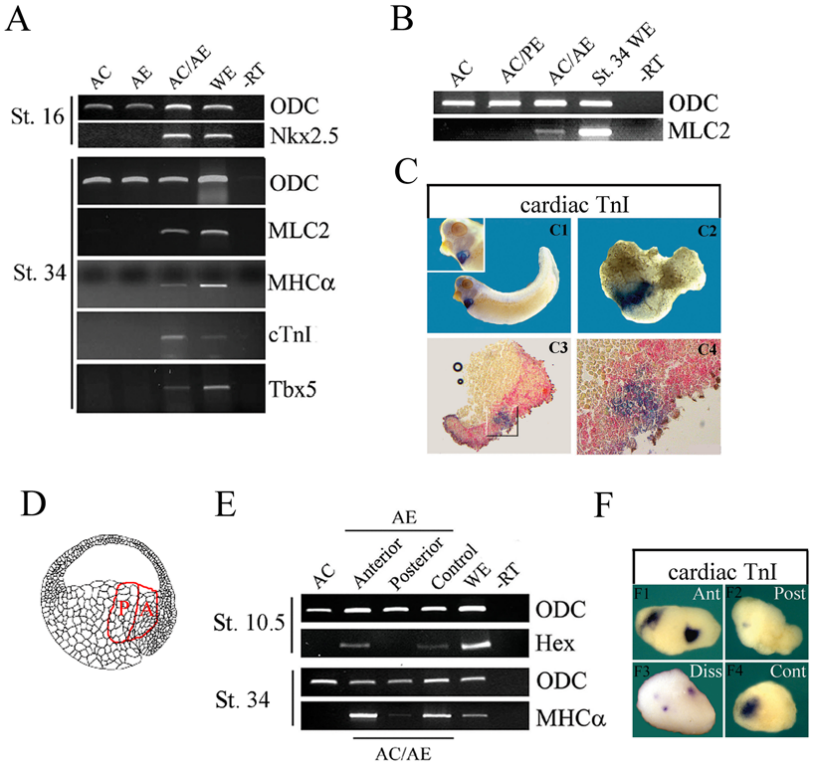
Anterior endoderm is sufficient to induce cardiogenesis in animal cap cells. Gene expression in explants were analyzed either by RT-PCR (A,B,E) or by in situ hybridization (C). (A) AC/AE conjugates express Nkx2.5 at st. 16, but animal caps and anterior endoderm alone do not. Cardiomyocyte-specific markers MLC2, MHCα and cardiac TnI, as well as cardiogenic transcription factor Tbx5 are all expressed in AC/AE conjugates at st. 34. (B) Posterior Endoderm (PE) is incapable of inducing cardiogenesis in animal caps. (C) Cardiac tissue (MLC2 expression; C1) is induced by anterior endoderm in animal cap·. Animal caps injected with biotinylated dextran were used to make conjugates with anterior endoderm, and after in situ hybridization for cardiac TnI (C2), lineage tracer was revealed by Fast Red staining on sections (C3,4). (D) A drawing of NF st. 10 embryo describing anterior endoderm explants and their subdivision into anterior (A) and posterior (P) parts. Source of drawing: www.xenbase.org. (E) Anterior half of anterior endoderm explants is greatly enriched in Hex expression (St. 10.5) and is a more efficient inducer of cardiogenesis in animal caps at st. 34. (F) Cardiac-inducing activity resides in the anterior (Ant) part of anterior endoderm explants. Double anterior endoderm/animal cap conjugates show two foci of cardiac TnI expression (F1), whereas double posterior endoderm/animal cap conjugates contain only few positive cells (F2). Dissociated and reaggregated anterior endoderm (Diss; F3) induces multiple smaller foci of cardiac gene expression in animal cap (in all 5 positive conjugates; n = 12). F4: control conjugates most frequently have a single cluster of cardiomyocytes (8/8 in this experiment, and 88% of all expressing explants overall, n = 74).

We devised a direct assay for cardiac specification to facilitate identification of cardiac-inducing signal(s) and in-depth analyses of the process. As early gastrula anterior endoderm is required for efficient cardiogenesis in *Xenopus* embryos and explants [2], we examined its ability to induce cardiac fate in pluripotent animal caps, which normally give rise to ectodermal derivatives and acquire an epidermal character when cultured alone. Anterior endoderm explants were dissected from st. 10 embryos and were shown to be free from mesoderm and Organizer tissue (Xbra^−^/Gsc^−^; Fig. S1) and were confirmed as anterior by detection of expression of anterior endoderm marker Hex (Fig. S1). Anterior endoderm explants were conjugated with animal caps (in 1∶3 ratio) and cardiac induction was assessed by expression of Nkx2.5, an early marker of heart field in neurula embryos, and at early tadpole stages by expression of a panel of cardiomyocyte-specific markers. As shown in Fig. 1A, anterior endoderm induced expression of Nkx2.5 in st. 16 conjugates and also of all myocardial-specific markers examined at early tadpole stages (MLC2, cTnI, MHCα). This was in contrast to posterior endoderm, which was incapable of inducing cardiogenesis (Fig. 1B). When cultured longer until st. 45 beating foci were seen occasionally in AC/AE conjugates (Movie S1), demonstrating physiological maturation of induced cardiomyocytes. Importantly, cardiogenesis in AC/AE explants occurred in the same temporal pattern as in sibling control embryos. This indicates that the AC/AE model faithfully recapitulates early heart development (Fig. S3).

As neither the anterior endoderm nor animal cap alone expressed cardiac markers, the induction of cardiac fate in AC/AE explants was caused by conjugation. We asked whether in AC/AE conjugates cardiac tissue is induced in animal cap by signals from anterior endoderm using in situ hybridization and lineage tracing. As shown in Fig. 1C, cardiac tissue is induced exclusively in the animal cap, usually in a single location adjacent to the inducer (71% have one locus and 11% have two closely linked loci of cardiac positive cells; the remaining 18% are negative (n = 74)). This observation suggested that the cardiac inducing signal is localized and non-uniform within the anterior endoderm explant. This hypothesis was confirmed by subdividing the anterior endoderm explant into the anterior and posterior halves. Two anterior and two posterior halves were used to make conjugates with animal caps, to compensate for the mass of endodermal tissue present in the inducer. This manipulation resulted in enrichment of cardiac inducing signal in the anterior-most endoderm containing conjugates, with very little activity remaining in explants made with the posterior part of inducing tissue (Fig. 1E). The increase in cardiogenesis could be attributed to more than one locus of cardiac tissue per conjugate (Fig. 1F). These results suggest that the cardiac-inducing signal is localized in the anterior-most region of endoderm, and that it acts locally. The cardiac-inducing capacity correlates with the Hex-expressing domain of anterior endoderm (Fig. 1E). Further evidence for discrete action of cardiac inducing signal was found using dissociated and re-aggregated anterior endoderm conjugated with animal caps. We found that cardiac tissue was induced in multiple small loci (Fig. 1F), suggesting that cardiac inducer tissue acts locally and proportionately. This also indicates that the cardiac-inducing signal(s) are either stably associated with the anterior endoderm cells or are continuously produced, as their inducing properties are not abolished by tissue dispersal.

### Anterior endoderm specifically induces cardiovascular cell fates

**Figure 2:**
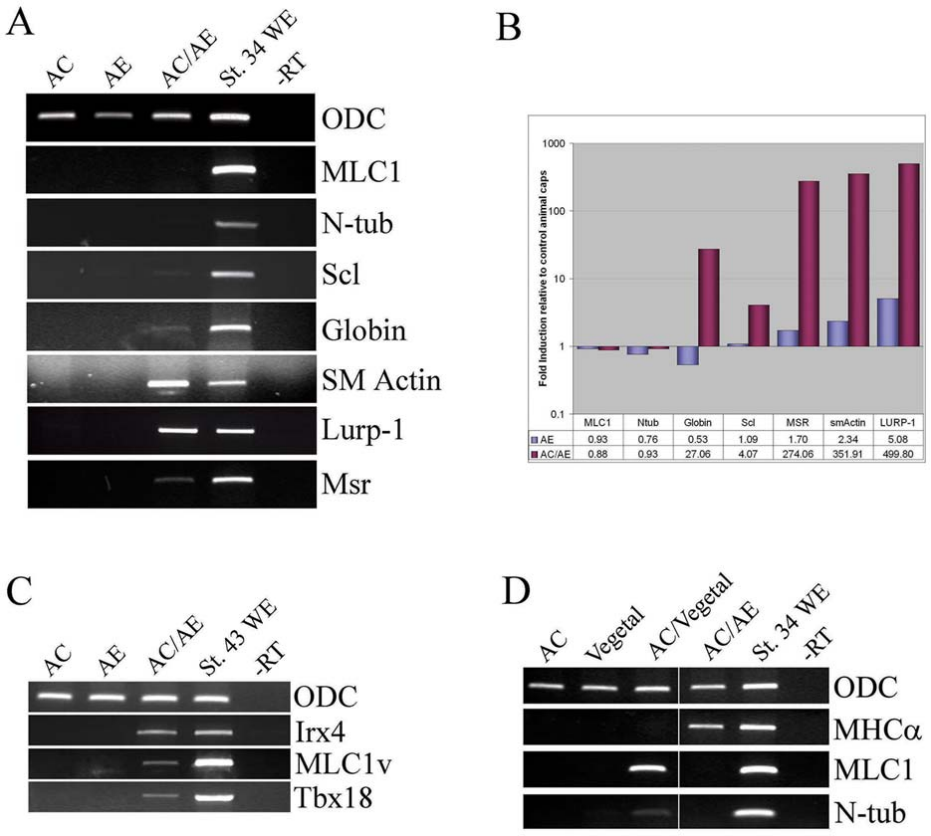
Specificity of anterior endoderm as inducer of cardiac fate. (A) anterior endoderm induces markers of cardiovascular cell types: blood (Scl, Globin), smooth muscle (SM actin), macrophages (Lurp-1) and endothelium (Msr), but not skeletal muscle (MLC1) or neural tissue (N-tubulin). (B) Quantification of RT-PCR shown in A. (C) Late tadpole-stage AC/AE explants express markers of ventricular myocardium Irx4 and MLC1v and of the proepicardium, Tbx18. (D) animal cap/vegetal pole conjugates (Nieuwkoop sandwiches) express skeletal muscle and neural, but not cardiac markers, in contrast to AC/AE conjugates.

In addition to cardiac tissue, anterior endoderm can induce markers of other cell types involved in cardiovascular development: endothelium, macrophages, smooth muscle, and blood (Fig. 2). Importantly, AC/AE explants were free from skeletal muscle (and neural tissue; Fig. 2; see also [50]), demonstrating that anterior endoderm is not inducing cardiac tissue as a part of general mesoderm induction, but is acting relatively specifically.

The presence of beating foci in the AC/AE conjugates suggested that the induced cardiac tissue had undergone a rudimentary functional organization and perhaps a degree of cellular diversification. At st. 43, AC/AE explants expressed ventricular markers MLC1v and Irx4 as well as the proepicardial marker Tbx18, suggesting that at least a limited extent of patterning of cardiac tissue has occurred (Fig. 2).

The AC/AE explants are heterochronic conjugates of st. 8.5 animal cap with st. 10 anterior endoderm and are a modification of Nieuwkoop sandwiches, which are isochronic conjugates of st. 8–8.5 vegetal poles and animal caps [51]. Classic Nieuwkoop conjugates result in robust induction of skeletal muscle and neural tissue [51], but significantly, cardiac tissue is not induced, in contrast to AC/AE conjugates (Fig. 2). This comparison demonstrates that the two inducing tissues produce distinct signals.

### Cardiac inducing signal requires 1–2 hours to specify cardiac fate

**Figure 3:**
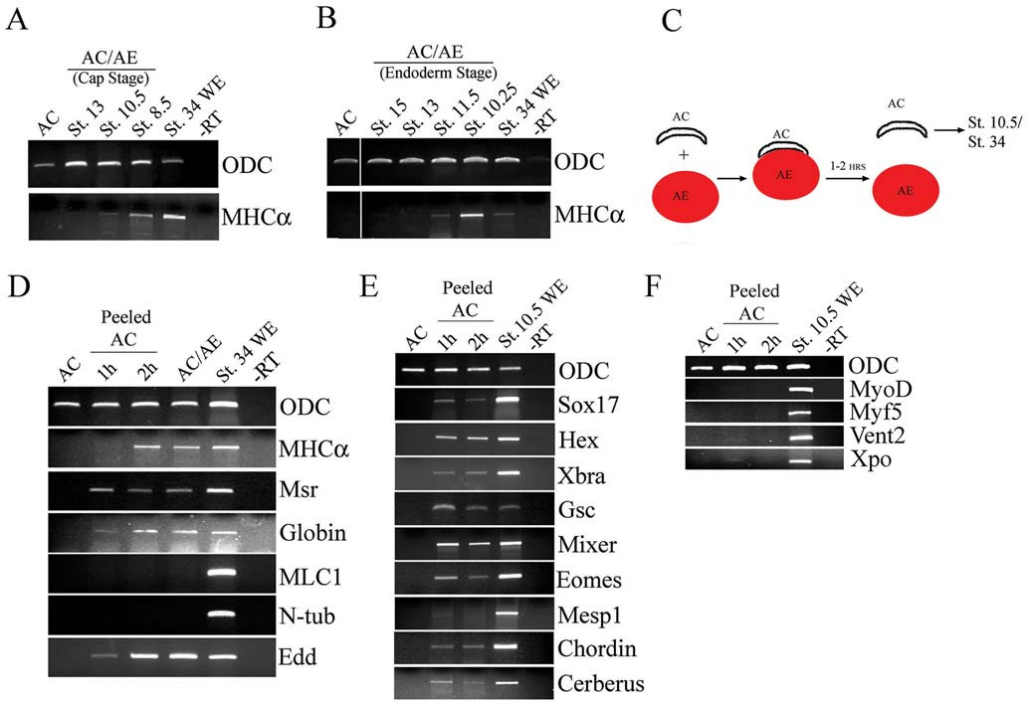
Two hours of contact between animal cap and anterior endoderm is sufficient for cardiogenesis. (A) Conjugates were made between st. 10.25 anterior endoderm explants and animal caps of aged animal caps isolated at st. 8.5, and MHCα expression was analyzed a day and a half later, at st. 34. (B) Conjugates were made between st. 8.5 animal caps and aged anterior endoderm explants isolated at st. 10, as indicated. MHCα expression was analyzed at st. 34. (C) Strategy for a peeled animal cap assay. Rhodamine-dextran injected anterior endoderm is conjugated with animal caps, and the caps are removed after 1 or 2 hours. Anterior endoderm-free (rhodamine-negative) animal caps are cultured until st. 34 or for a total of 2.5 hours after the initial contact, corresponding to st. 10.5 (D) Only animal caps that were in contact with anterior endoderm for 2 hours express MHCα, but other markers tested – Msr, Globin, Edd – are also induced after 1 hour of contact. (E) Early markers of endoderm (Sox17, Hex, Cerberus, Mixer), Organizer (Goosecoid (Gsc), Chordin) and mesoderm (Xbra, Eomesodermin) are expressed after 1 hour contact with anterior endoderm. However Mesp1, a marker of migrating cardiac precursors [62], was not induced. (F) Myogenic regulatory factors MyoD and Myf5 as well as posterior markers Xpo and Vent2 are not induced by AE.

In order to establish the time at which the animal caps can respond to anterior endoderm-derived signal, we made conjugates with animal cap explants that were excised at st. 8.5 and then aged in culture. The animal caps gradually lost competence to respond to cardiac fate-inducing signal from anterior endoderm, so that by st. 13 very little cardiac tissue was induced (Fig. 3). Similarly, the duration of the anterior endoderm-derived signal was limited to mid-gastrulation (Fig. 3). Having established that cardiac induction in AC/AE explants can occur efficiently during the narrow time window following conjugation, we wished to precisely define the time when induction actually takes place. In order to accomplish this, we adopted an assay for timed exposure of animal cap to anterior endoderm, in which we removed or peeled animal cap from anterior endoderm after defined time. The basic design of this assay was described by Gurdon and colleagues in their studies of muscle induction [52]. In our modification we labeled anterior endoderm with rhodamine-dextran to ensure that no anterior endoderm cells remained on animal cap explants after peeling (Fig. 3). Using this assay we found that induction of cardiomyocyte markers in animal caps required only a 2 hour exposure to anterior endoderm and that 1 hour was insufficient. The overall profile of cellular fates induced in peeled animal cap after 2 hours of exposure was indistinguishable from AC/AE conjugates (Fig. 3), suggesting that in this model system 2 hours of interaction between the inducer and the responder is sufficient to induce a range of cardiovascular cell types.

Our results show that a limited range of cellular fates are induced in AC/AE conjugates. We next asked whether known early targets of mesendoderm induction are also selectively induced. Animal cap explants were peeled after 1 or 2 hours of exposure to anterior endoderm and collected 2.5 hours after initiation of contact. Analysis of a panel of mesendodermal genes revealed that the markers of mesoderm (Xbra and Eomesodermin), endoderm (Sox17, Hex, Mixer and Cerberus) and the Organizer (Goosecoid and Chordin) were all induced (Fig. 3). In contrast, animal caps exposed to anterior endoderm did not express the myogenic regulatory factors Myf5 and MyoD, demonstrating that myogenic differentiation, which is not detected at tadpole stages (no MLC1 expression; Fig. 2), is not initiated (Fig. 3). Posterior markers Xpo and Vent2 were also absent, suggesting that the signal is selective for anterior mesendoderm fates (Fig. 3).

Interestingly, tested mesendodermal genes were expressed equally after 1- or 2 hours of exposure, a pattern that did not correlate with the induction of cardiac markers only after 2 hours of exposure of animal cap to anterior endoderm. This finding most likely reflects the current ignorance of early markers of cardiomyocyte fate.

Our results show that anterior endoderm induces both endodermal and mesodermal markers in animal caps (Fig. 3) and raise the possibility that the induced endoderm might influence cardiogenesis. Conjugates in which animal cap explants were expressing dominant-negative Sox17β protein induced higher level of cardiac markers (Figs. S2, S4), suggesting that in AC/AE explants, Sox17-dependent endoderm opposes cardiogenesis; a similar result was previously described in animal caps in which mesoderm and endoderm were induced by GATA4 [44].

Having established some of the basic characteristics of the cardiac inducing signal we then extended the investigation of the nature of the signal by manipulating signalling capacity of the responder. In the following experiments our objective was to modulate the signalling pathways that may be involved in the interpretation of the signal while avoiding interference with the production of the signal, as this would likely also affect normal germ layer formation and patterning of the embryonic axes with the potential to influence cardiogenesis indirectly. Cardiac differentiation was scored in these experiments at early tadpole stages (st. 34), when cardiomyocyte-specific markers are expressed robustly, but prior to the advanced stages of heart growth and morphogenesis.

### FGF and Nodal signaling are required during the first hour of cardiac induction

**Figure 4:**
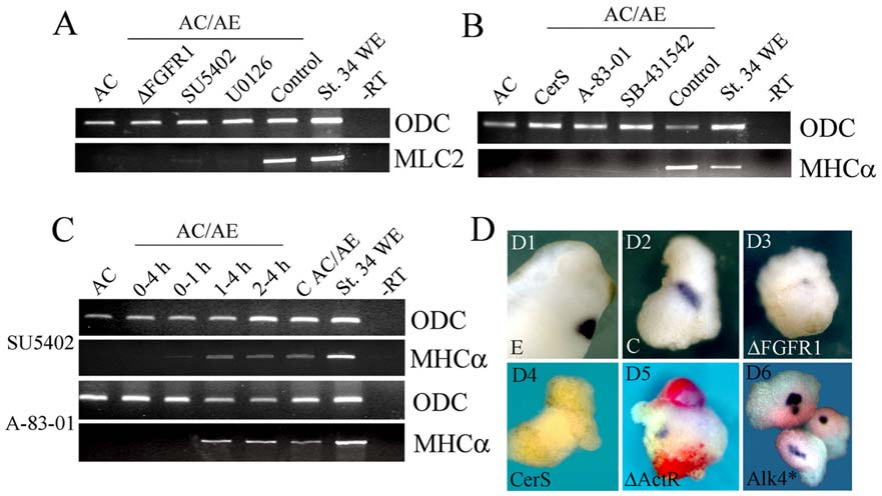
FGF and Nodal signaling are required during the first hour of cardiac induction. (A) AC/AE conjugates were made with animal cap expressing ΔFGFR1 or with uninjected animal cap that were continuously incubated with SU5402 (50 µM) or U0126 (35 µM) from the time of conjugation, or were treated with DMSO (Control). MLC2 expression was analyzed at st. 34. (B) CerS (injected in animal cap) or continuous incubation with SB-431542 (75 µM) or A-83-01 (75 µM) block induction of MHCα. (C) FGF and Nodal signaling are only required the first hour of contact between AC and AE. (D) In situ hybridisation analysis of cardiac TnI expression in AC/AE conjugates. D1,2- control embryo and AC/AE explant. 83% (n = 23) explants are cTnI+. D3- expression of ΔFGFR1 in animal caps leads to a greatly reduced expression of cTnI in 89% (n = 19) explants. D4- 0/15 of CerS explants express cTnI. D5- mosaic expression of ΔActRI (red) in animal caps prevents expression of cardiac TnI (0/11 cTnI+ explants in ΔActRI-injected cells), but not in neighboring tissue with intact Nodal signaling (5/11 cTnI+ explants; arrow in D5). D6- constitutively active Alk4* receptor induces cardiac expression cell-autonomously in animal caps (4/4 cTnI+, n = 12).

FGF and Nodal signaling have been implicated in cardiac specification. We explored the requirement for FGF signaling in the AC/AE model using either overexpression of dominant-negative FGF receptor 1, ΔFGFR1, or treatment of AC/AE explants with a soluble inhibitor of FGFRs (SU5402; [29]) from the time of conjugation. Both treatments strongly reduced the level of the cardiac marker MLC2 expression (Fig. 4A). A similar result was obtained by inhibition of ERK, a downstream mediator of FGF signaling, with soluble inhibitor U0126 (Fig. 4A). The role of Nodal signaling in AC/AE explants was examined using three specific inhibitors: Cerberus-Short, a Nodal-specific domain of secreted factor Cerberus [38], [39], and two soluble drugs, SB-431542 [27] and A-83-01 [28]. All three reagents efficiently blocked cardiogenesis in AC/AE explants (Fig. 4B). We used SU5402 and A-083-01 to determine the length of time during which FGF and Nodal signaling, respectively, are required for cardiogenesis. Initially we used two treatment windows, during or after gastrulation. This experiment revealed that FGF and Nodal signalling are required only during gastrulation (Fig. S5). We then focused on the first 4 hours of incubation (corresponding approximately to st. 11). A brief treatment during the first hour of conjugation was sufficient to block cardiogenesis (Fig. 4C). In contrast, SU5402 or A-83-01 treatment after 1 hour of contact had no obvious effects (Fig. 4C). We have confirmed these results using WMISH showing inhibition of expression of for cardiac TnI in AC/AE conjugates prepared with animal cap explants expressing ΔFGFR1 or Cer-S (Fig. 4D).

We also examined cell autonomy of Nodal action by analyzing cardiogenesis in conjugates in which one half of an animal cap was expressing dominant-negative activin receptor ΔActRI. ΔActRI inhibits both activin/Nodal and BMP signaling [41], however since BMP signaling is not required for cardiogenesis (see below) in these experiments this reagent is used as cell-autonomous inhibitor of Nodal signaling. In such conjugates cardiac tissue is induced in the animal cap only when activin/Nodal signaling is intact (Fig. 4D). Therefore, activin/Nodal signaling was required cell-autonomously to induce cardiac tissue. Activin/Nodal signaling is also sufficient for cell-autonomous induction, as constitutively activated Alk4 receptor induced cardiogenesis in animal caps (Fig. 4D).

### ERK activity is required for at least the first 4 hours of cardiac induction

**Figure 5:**
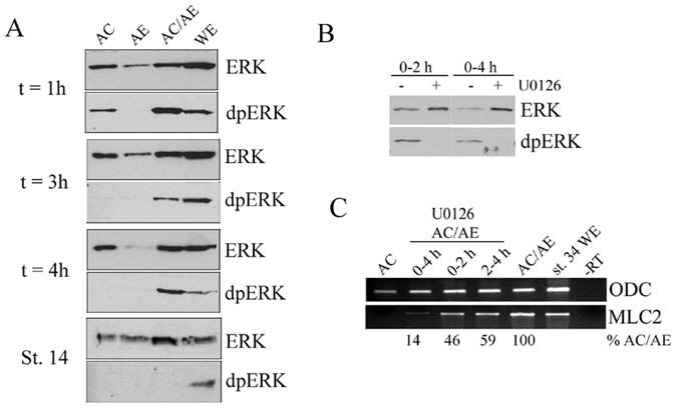
ERK is activated during the first 4 hrs and is required for cardiogenesis over the same period. (A) ERK is activated for at least 4 hours after conjugation of animal cap and anterior endoderm. Animal cap, anterior endoderm or AC/AE explants were collected at indicated times after conjugation and were subjected to Western analysis for doubly-phosphorylated ERK (dpERK) and total ERK. The latest time-point (St. 14) corresponds to 8–10 hours after conjugation. (B) U0126 treatment for the first 2 or 4 hours after conjugation effectively blocks ERK activation. (C) ERK activity is required during at least first four hours after conjugation for efficient cardiogenesis.

The activation of ERK in AC/AE explants was demonstrated by detecting phosphorylated form of ERK (Fig. 5A). A transient activation of ERK accompanies excision of animal caps [53], [54], which prevented us from establishing the precise time of induction of ERK by conjugation (Fig. 5A). Nonetheless, activated ERK was detectable in conjugates throughout the first four hours of induction, demonstrating that conjugation of animal caps with anterior endoderm induces sustained ERK activity. Treatment of AC/AE explants with U0126 during the same period (first 4 hours of conjugation) inhibited ERK activation (Fig. 5B) and substantially reduced cardiogenesis (Fig. 5C). A subdivision of the 4 hour window into two shorter periods (0–2 and 2–4 hours) of treatment with U0126 reduced MLC2 expression by a small amount (Fig. 5C). Two hours of ERK activity during the first 4 hours of induction is sufficient for cardiogenesis that is half as efficient as in control AC/AE conjugates (Fig. 5C). One implication of this result is that ERK activity is required during the entire 4 hour period for maximal cardiogenesis (relative to control AC/AE explants), in contrast to an absolute requirement of FGF ligand-receptor interaction in the first hour of induction.

### BMP signaling is not required for cardiac specification in AC/AE explants

**Figure 6:**
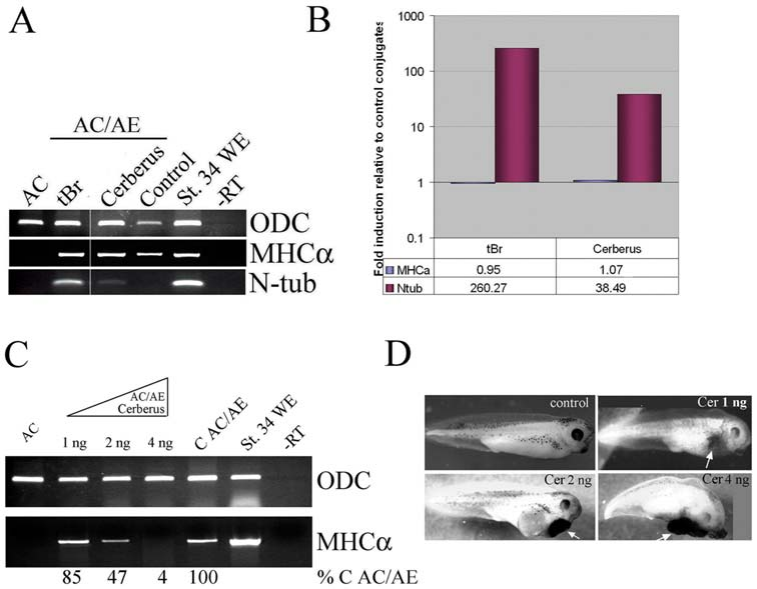
BMP signaling is not required for cardiogenesis in AC/AE model. (A) Animal caps expressing truncated type I BMP receptor (tBR) or Cerberus (1 ng of mRNA each), were conjugated with anterior endoderm and conjugates were analyzed for expression of MHCα and N-tubulin at st. 34 by RT-PCR. (B) Quantification of RT-PCR in A. Both anti-BMP reagents used were active, as shown by induction of N-tubulin expression, but had no effect on cardiogenesis. (C) Dose-dependent inhibition of cardiogenesis in AC/AE explants by Cerberus. Animal caps from embryos injected with 1, 2 or 4 ng of Cerberus mRNA were conjugated with anterior endoderm and conjugates were analyzed for MHCα expression when sibling control embryos reached st. 34. The effect of Cerberus was quantified and normalized to control conjugates (% C AC/AE). (D) Phenotypic controls for Cerberus mRNA, showing dose-dependent increase in cement gland tissue.

We tested the requirement for BMP signaling for cardiogenesis in AC/AE explants using a dominant-negative BMP receptor type I and Cerberus, a multivalent inhibitor of BMP, Nodal and Wnt signaling. The effectiveness of these reagents was demonstrated by induction of neural tissue in animal caps (Fig. 6). However, they had no effect on cardiogenesis (Fig. 6), demonstrating that BMP signaling is not required for cardiogenesis in the AC/AE model system, at least during the early phases of differentiation. Cerberus displayed dose-dependent action, as it blocked BMP signaling but not cardiogenesis at 1 ng, while higher concentrations also inhibited cardiogenesis (Fig. 6), similar to the effect of approximately equimolar Cer-S (Fig. 4).

### Dkk-1 enhances cardiogenesis

**Figure 7:**
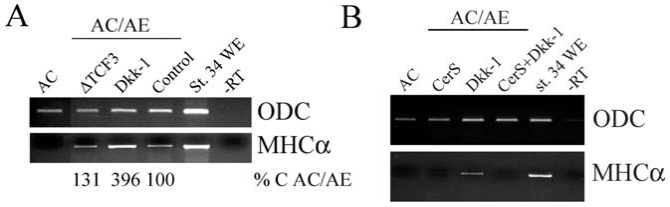
Dkk-1 enhances cardiogenesis. (A) animal cap injected with 0.5 ng of ΔTcf3 or 1 ng of Dkk-1, or uninjected control animal cap, were conjugated with anterior endoderm and analyzed for MHCα expression. The effect of Dkk-1 was quantified and shown below the image. In two additional experiments, Dkk-1 stimulated cardiogenesis 5- and 9-fold. (B) Dkk-1 cannot act in Nodal-independent manner, as it is blocked by CerS. The effectiveness of ΔTcf3 was shown by posteriorization of embryos (data not shown).

Inhibition of the Wnt/β-catenin signaling pathway stimulates cardiogenesis in chick and *Xenopus* lateral plate/marginal zone explants [22], [23] and in GATA4-injected animal caps from *Xenopus* embryos [44]. In agreement with these studies, we found that in AC/AE explants, Dkk-1 stimulates cardiogenesis (Fig. 7A). Interestingly, stimulation of cardiogenesis appeared not to be shared by other Wnt antagonists, as a dominant-negative TCF3 (ΔTCF3) construct had no such effect (Fig. 7A). Others have reported that Dkk-1 might enhance cardiogenesis through both Wnt/β-catenin dependent and independent modes [55]. Nonetheless, at present we cannot exclude a role for Wnt antagonism in cardiogenesis. Importantly, however, Dkk-1 is unlikely to act instructively as it cannot induce cardiogenesis in AC/AE conjugates independently of the Nodal pathway (Fig. 7B).

### Wnt/b˜catenin signaling inhibits cardiogenesis after specification

**Figure 8:**
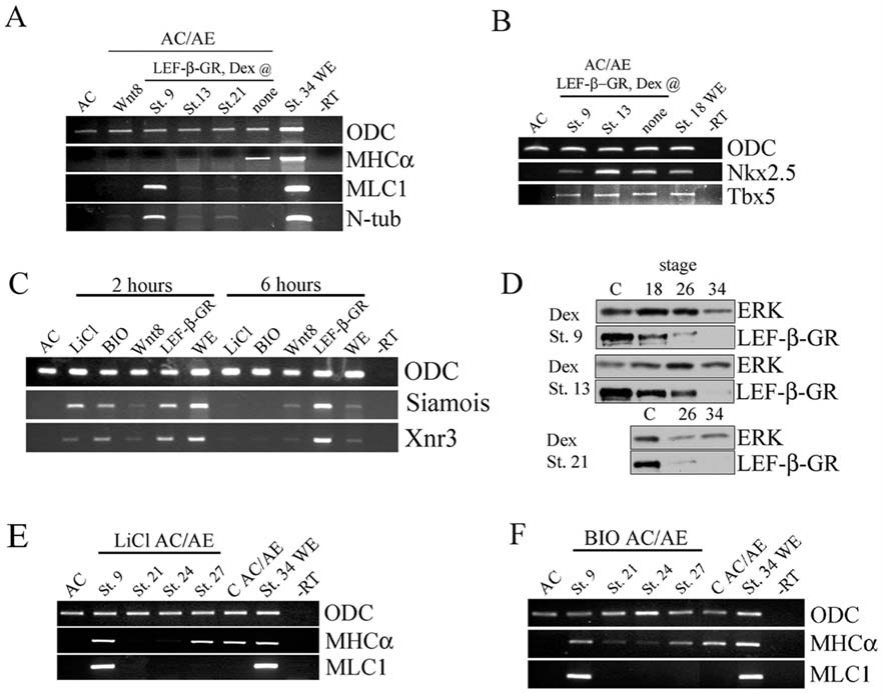
Elevated Wnt/β-catenin signaling opposes cardiogenesis after specification. (A) Animal cap expressing either CSKA-Wnt8 or LEF-β-catenin-GR were conjugated and dexamethasone was added as indicated. All treatments block cardiogenesis. (B) LEF-β-GR activated at st. 9 and st. 13 has no effect on Nkx2.5 and Tbx5 expression at st. 18. (C) The effect of various agonists on the Wnt/β-catenin pathway: LiCl and BIO cause strong transient activation, Wnt8 causes weak and sustained activation, and LEF-β-catenin-GR causes strong and prolonged effect. Siamois and Xnr3 expression was analysed 2 and 6 hours after excision of animal caps/Treatment with LiCl, BIO and Dex was immediately after the excision. (D) The expression of the LEF-β-catenin-GR fusion protein was analyzed by Western blotting for HA tag. The protein was expressed efficiently at all stages at which dexamethasone was added and it persists for many hours. (E, F) LiCl or BIO treatment at st. 9 has no effect on cardiac differentiation but induces skeletal muscle. Later treatment at st. 21 or 24 blocks cardiac differentiation, and treatment at st. 27 has no effect.

We examined the consequences for cardiogenesis of elevated levels of Wnt/β-catenin signaling, by manipulating the pathway by DNA-mediated overexpression of Wnt-8 and by inducible activation of Lef-β-catenin-GR fusion protein, a potent transcriptional activator [40]. Both CSKA-Wnt8 and Lef-β-catenin-GR activated at the time of conjugation (st. 9) efficiently blocked cardiogenesis, suggesting that elevated levels of Wnt/β-catenin signaling are not compatible with cardiac specification (Fig. 8A). We have used the inducibility of Lef-β-catenin-GR by dexamethasone to determine the effect of increased Wnt/β-catenin signaling at later stages of cardiogenesis. Activation of Wnt/β-catenin pathway after gastrulation, at st. 13 or 21, also prevents cardiac differentiation (Fig. 8A) but with no effect on specification, as assessed by Nkx2.5 and Tbx5 expression (Fig. 8B). Even though both CSKA-Wnt8 and Lef-β-catenin-GR inhibited cardiogenesis, they had differential effect on myogenesis. Lef-β-catenin-GR, but not CSKA-Wnt8, can induce skeletal muscle in AC/AE conjugates when activated at st. 9 (Fig. 8B). The difference between these two modes of activation of Wnt/β-catenin signaling likely reflects their differential effectiveness. Using the CSKA promoter results in mosaic expression and it likely takes several hours to accumulate sufficient exogenous Wnt8 protein to activate Wnt pathway. As expected, CSKA-Wnt8 induced sustained low-moderate levels of expression of direct targets of Wnt pathway, Siamois and Xnr3 (Fig. 8C). In contrast, Lef-β-catenin-GR protein can be activated very efficiently by dexamethasone at will, leading to strong and lasting activation of Siamois and Xnr3 (Fig. 8C). This finding suggests that myogenesis requires relatively high level of Wnt/β-catenin signaling in the AC/AE model.

A Western blot for HA-tagged Lef-β-catenin-GR in explant samples collected at the time of dexamethasone addition showed that the fusion protein was present at approximately equal levels (Fig. 8D), and that the induced Lef-β-catenin-GR protein persists (Fig. 8D) and acts to induce its target genes for many hours (Fig. 8C). To more precisely determine when Wnt/β-catenin signaling blocks cardiogenesis, we used soluble drugs LiCl and BIO to transiently stimulate the pathway at different time points. Transient effect of LiCl and BIO was confirmed by detection of Siamois and Xnr3 at 2 hours but not after 6 hours following the treatment (Fig. 8C). In contrast, Lef-β-catenin-GR strongly stimulated Siamois and Xnr3 both at 2 and 6 hours after activation by dexamethasone (Fig. 8C). Early LiCl treatment at the time of conjugation had no effect on cardiogenesis, while it stimulated myogenesis (Fig. 8E). In contrast, LiCl treatments at st. 21 and 24 blocked cardiac differentiation (Fig. 8E). Once cardiac differentiation commences (st. 27), LiCl had no effect (Fig. 8E). The effects of LiCl were mirrored by BIO, confirming their specificity (Fig. 8F). We conclude that Wnt/β-catenin signaling has no effect on cardiac specification and that it antagonizes cardiogenesis prior to the onset of differentiation.

## Discussion

Anterior endoderm has been known to play a key role in cardiac induction in vertebrates; the approach described here provides a direct assay for investigating the details of this role and of cardiac specification. The assay shows that the cardiac inducing signal generated by anterior endoderm requires up to two hours for specifying cardiac fate by activation of FGF and Nodal pathways. The signal provided by anterior endoderm is distinct from general mesendoderm inducing signal generated by blastula stage vegetal pole, as skeletal muscle is not induced. Similarly, early genes induced by the signal include anterior, but not posterior mesendodermal markers. Even though several endodermal and Organizer markers are induced, we believe that cardiac induction in the AC/AE model occurs directly and not subsequently via signaling sources induced in the responding tissue. We have directly ruled out a role for Sox17-dependent endoderm in cardiogenesis in the responding tissue (Figs. S2, S4). Additional evidence showing Wnt-independent cardiogenesis in explants (Fig. 8) makes the involvement of putative Wnt-dependent signaling centers such as the Organizer unlikely. Further results suggest that FGF and Nodal pathways are only needed during the first hour of contact between the inducer and the responder, making it improbable that a FGF- and Nodal-dependent secondary signaling center is generated in the responding tissue.

An important difference between our findings and previous studies is that using the AC/AE model we can explore directly the proximal events of cardiac induction. Unlike systems such as ventral marginal zone explants from *Xenopus* embryos and lateral or posterior mesoderm explants from chick embryos, the AC/AE model does not rely on modification of pre-existing posterior mesoderm, as cardiac mesoderm is induced de novo by the signals from the relevant source, gastrula-stage anterior endoderm.

The assay employs conjugation of heterochronic endodermal and ectodermal explants, which makes a direct study of induction feasible even though this interaction does not normally occur. Nonetheless, given that AC/AE conjugates faithfully reproduce virtually every aspect of early cardiogenesis, they are a physiologically relevant and tractable model for detailed analysis of cardiac induction.

The current study has shown that FGF is essential for cardiac induction in *Xenopus.* Previous work has implicated FGF signaling in cardiac specification in zebrafish and chick embryos, but little is known about the directness and cardiac specificity of this role [11]–[15]. We have complemented those studies by providing evidence for a specific and direct role of FGF signaling in cardiac induction.

Similarly, Nodal signaling has been suggested to play a role in cardiac specification either indirectly, through genetic analyses in zebrafish and mouse [8], [16]–[18] or in in vitro models [19], [20]. We have extended these findings by showing that Nodal likely acts directly as an essential component of the anterior endoderm-derived cardiogenic signal.

Our findings identify an essential role for FGF and Nodal signaling during the first hour of cardiac induction. We suggest they are required simultaneously or in a very rapid sequence, in apparent contrast to the relationship of these factors in the induction of mesoderm, which is initiated by activin/Nodal signaling and maintained by FGF signaling by a mechanism including positive feedback [54], [56], [57]. The response of animal cap cells to FGF can be modulated by TGFβ, leading to synergistic induction of muscle [58]. More recently, an interaction between FGF and activin, mediated by phosphorylation of p53, has been implicated in mesoderm specification in *Xenopus* [59], but a cardiac role for this mode of regulation has not been investigated. Our next task will be to establish how the FGF and Nodal pathways act in cardiac induction; once this is achieved it will be possible to reconstitute the cardiac inducing signal and to demonstrate the sufficiency of these pathways for mediating cardiac specification.

We considered which FGF and Nodal factors might be required for cardiogenesis. Multiple members of the FGF and Nodal families are known to be expressed in early gastrula anterior endoderm, and in our incomplete survey we detected Xnr1, 2 and 5 as well as FGF3, 4, 9 and 20 (Fig. S6). The scenario of considerable redundancy and complexity within both Nodal and FGF families present in the anterior endoderm indicates that defining cardiac-inducing roles for these factors will be a challenging task.

In contrast to the role of FGF receptor signaling –which is only required during the initial hour of cardiac induction– we found that cardiac induction needs sustained activation of ERK over at least four hours. In cell culture models, the duration of ERK activity induced by diverse stimuli is known to control a choice of physiological responses (e.g. differentiation or proliferation; [60]). It will be of considerable interest to reveal the mechanism by which embryonic cells interpret sustained ERK activity in the context of the cardiac specification program.

We found that transient activation of Wnt/β-catenin pathway during gastrulation has no effect on cardiogenesis, whereas more sustained or later activation of the pathway blocks differentiation of cardiomyocytes without affecting their specification. Our results show a requirement for suppression, or low activity, of Wnt/β-catenin pathway during a period of several hours just prior to the onset of cadiomyocyte differentiation. Previous work using chick and *Xenopus* embryos and explants has shown that overexpression of Wnt3 or Wnt8 inhibits cardiogenesis [22], [23], and this result is comparable to our finding with DNA-mediated overexpression of Wnt8 (Fig. 8). In these experiments, the time window of sensitivity of cardiogenesis to the Wnt/β-catenin pathway could not be determined because of sustained activation of the pathway.

Our results are in agreement with those showing Wnt-mediated suppression of cardiogenesis after specification obtained in ES cells [24], [25]. One difference between our model and ES cells is that we find no evidence for stimulation of cardiac specification by Wnt/β-catenin pathway. This is likely because in our assay, the inducer, anterior endoderm, has been generated by Wnt/β-catenin pathway through its well-established role in the establishment of dorso-ventral axis, whereas in ES cells cardiac inducing signals have to be generated by a process that likely mimics at least some aspects of germ layer specification that require Wnt/β-catenin pathway.

Our findings can be summarized as follows: early embryonic cells that have experienced Nodal and FGF signals and are briefly exposed to Wnt/β-catenin signaling can adopt both skeletal and cardiac muscle fates. Subsequent exposure of specified precursors to Wnt/β-catenin signaling blocks cardiac differentiation, via a mechanism that is presently unknown, but is unlikely to involve either a selective removal of specified precursors (Fig. 8), or a block of proliferation [61].

## Supporting Information

Figure S1 Anterior endoderm explants from early gastrula embryos are Xbra-/Gsc-/Hex+. Whole embryos (WE), Dorsal- or Ventral Marginal Zones (DMZ or VMZ) and AE (anterior endoderm) from st 10-10.2.5 embryos and AC (animal cap) from st. 8.5–9 embryos were analyzed by RT-PCR for expression of ODC (A), Hex (B), Goosecoid (C) and Xbra (D). Samples were taken after 25, 28, 32 and 35 cycles to ensure sensitivity and linearity of detection. (E) Quantification of A–D.
(7.24 MB TIF)

Figure S2 Linearity of gene expression detection by gel RT-PCR for markers used in this study. (A) St. 34 embryo sample was used. (B) Quantification of the data in (A). (C, D) Comparison of RT-PCR and qPCR detection of enhancement of expression of MLC2 by expression of 250 pg of Sox17beta-Engrailed Repressor (ER) protein in animal caps of AC/AE conjugates. In two repeats, Sox17beta-ER stimulated cardiogenesis 7- and 10-fold.
(10.23 MB TIF)

Figure S3 Cardiogenesis in AC/AE conjugates occurs at the same time as in sibling control embryos. AC/AE and sibling whole embryo (WE) samples were collected at st.18, 24, 27 and 34 and were analyzed for expression of indicated markers by RT-PCR.
(1.04 MB TIF)

Figure S4 Sox17-dependent endoderm in responding tissue is not required for cardiogenesis in AC/AE model. (A) Animal caps from embryos injected with Sox17beta-Engrailed Repressor (Sox17ER) were conjugated with anterior endoderm and analyzed for MHCalpha expression when sibling control embryos reached st. 34. (B) Quantification of a gel in A, confirming that Sox17ER enhances cardiogenesis.
(1.76 MB TIF)

Figure S5 FGF and Nodal signalling are required from st. 9-st.12 (9–12), but not after st. 12 (12–34).
(2.22 MB TIF)

Figure S6 Anterior endoderm explants express several Nodal and FGF genes. Anterior endoderm explants were analyzed for expression of indicated genes immediately after excision. All genes tested are expressed in st. 10 anterior endoderm. Anterior character of endoderm explants was confirmed by expression of Cerberus and Hex.
(1.25 MB TIF)

Table S1 Information about the primers used in the study.
(0.13 MB DOC)

Movie S1 St. 45 AC/AE explants show rhythmic beating, demonstrating physiological maturation of induced cardiomyocytes.
(0.84 MB WMV)
