# Supplementary figures and images for: Early Activation of FGF and Nodal Pathways Mediates Cardiac Specification Independently of Wnt/β-Catenin Signaling
Source: PLoS One. 2009 Oct 28;4(10):e7650. doi: 10.1371/journal.pone.0007650 (PMC2763344; doi:10.1371/journal.pone.0007650)

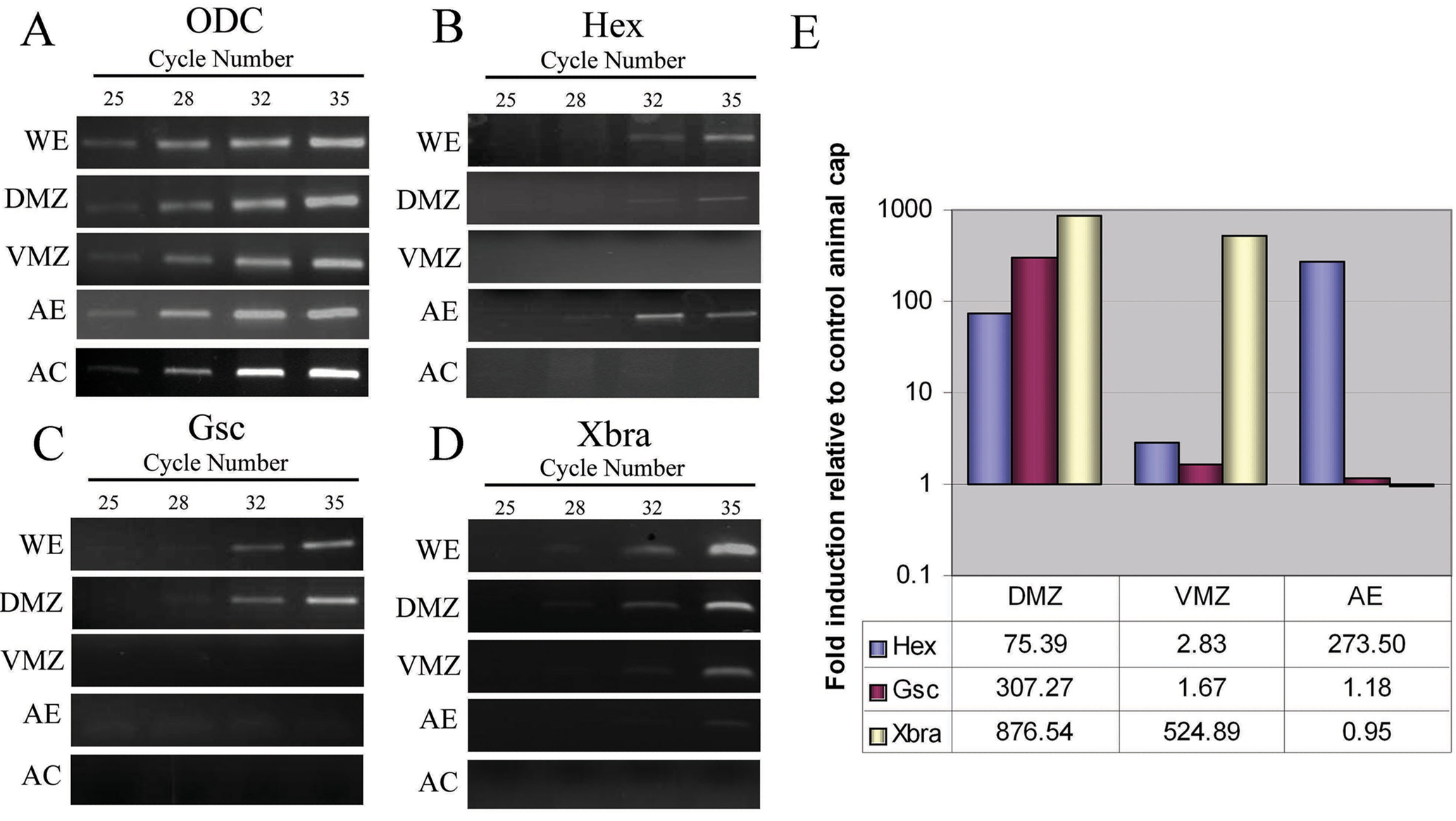

Supplement: Figure S1 — Anterior endoderm explants from early gastrula embryos are Xbra-/Gsc-/Hex+. Whole embryos (WE), Dorsal- or Ventral Marginal Zones (DMZ or VMZ) and AE (anterior endoderm) from st 10-10.2.5 embryos and AC (animal cap) from st. 8.5–9 embryos were analyzed by RT-PCR for expression of ODC (A), Hex (B), Goosecoid (C) and Xbra (D). Samples were taken after 25, 28, 32 and 35 cycles to ensure sensitivity and linearity of detection. (E) Quantification of A–D. (7.24 MB TIF) [file pone.0007650.s001.tif]

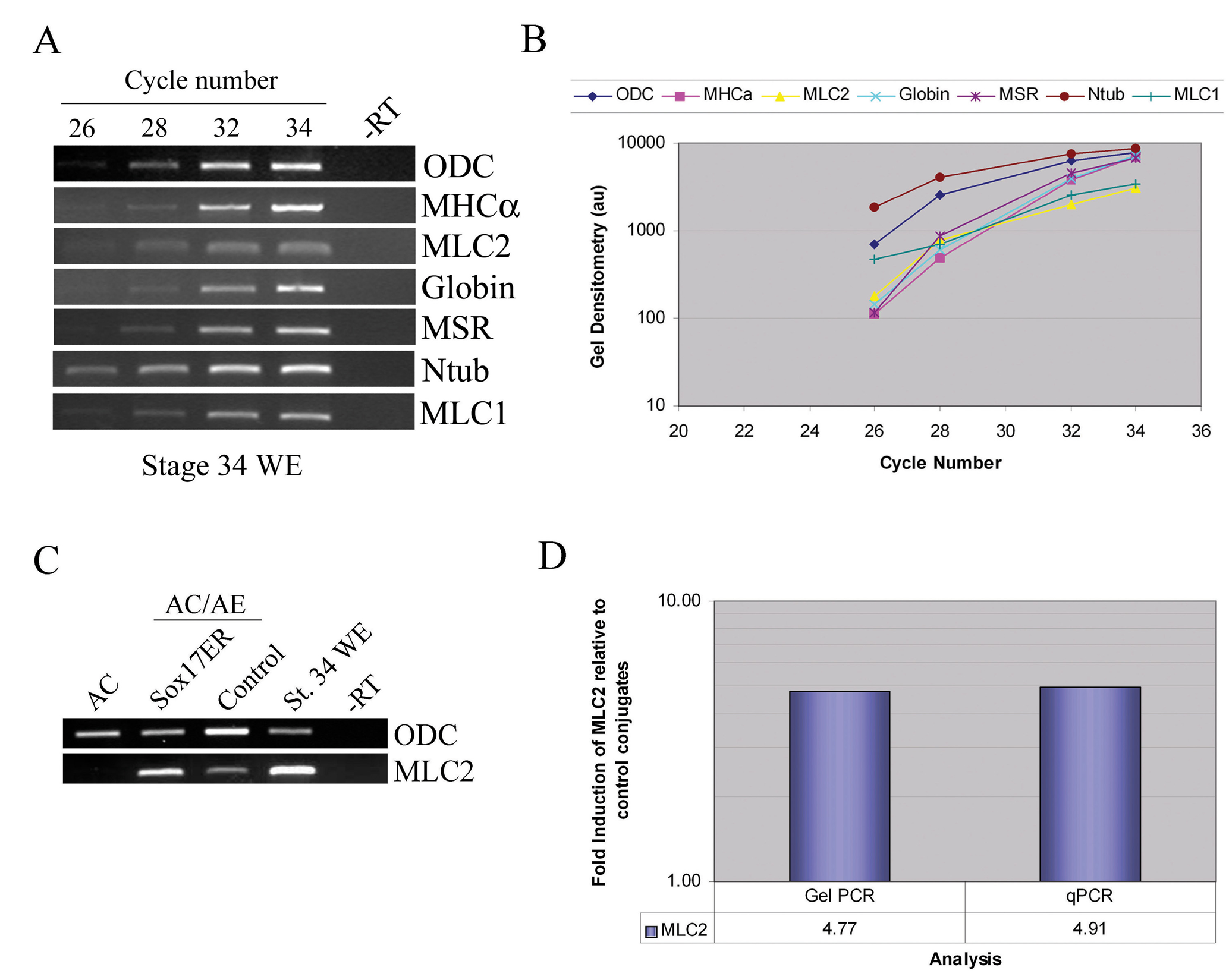

Supplement: Figure S2 — Linearity of gene expression detection by gel RT-PCR for markers used in this study. (A) St. 34 embryo sample was used. (B) Quantification of the data in (A). (C, D) Comparison of RT-PCR and qPCR detection of enhancement of expression of MLC2 by expression of 250 pg of Sox17beta-Engrailed Repressor (ER) protein in animal caps of AC/AE conjugates. In two repeats, Sox17beta-ER stimulated cardiogenesis 7- and 10-fold. (10.23 MB TIF) [file pone.0007650.s002.tif]

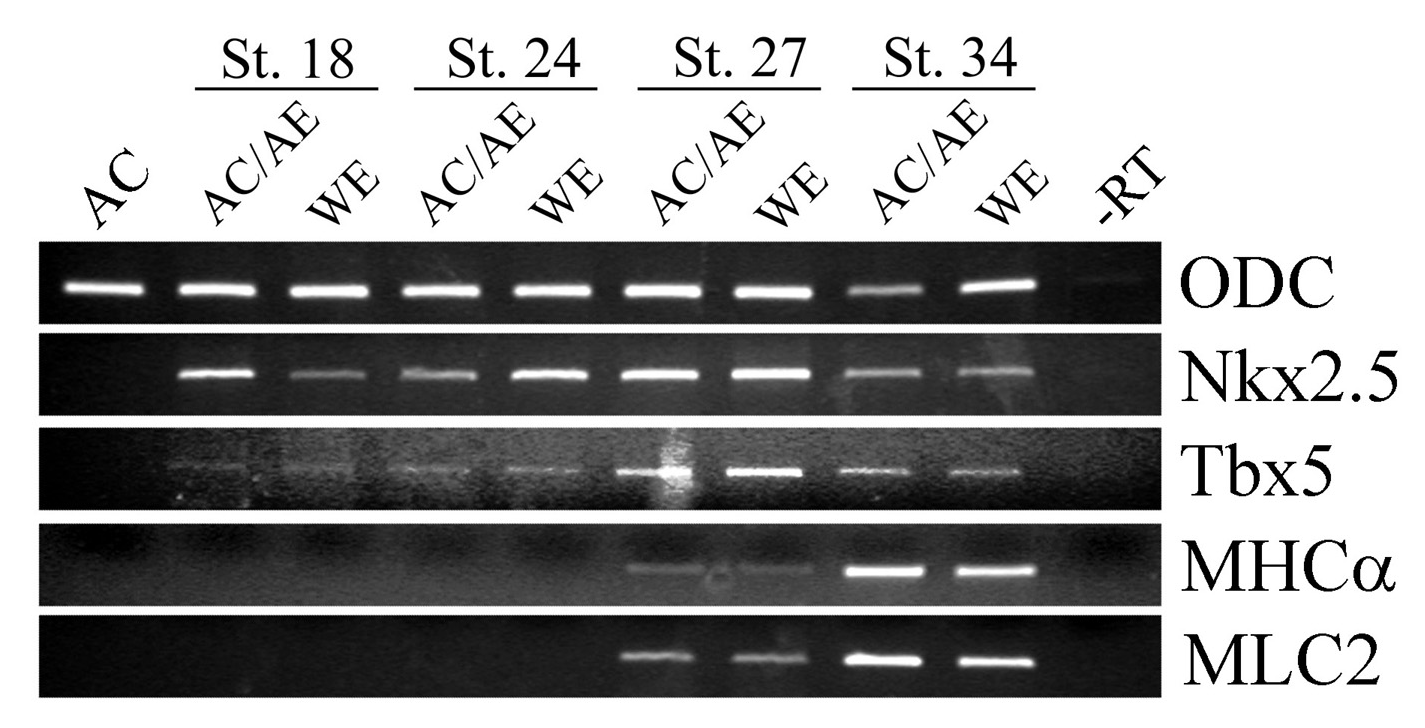

Supplement: Figure S3 — Cardiogenesis in AC/AE conjugates occurs at the same time as in sibling control embryos. AC/AE and sibling whole embryo (WE) samples were collected at st.18, 24, 27 and 34 and were analyzed for expression of indicated markers by RT-PCR. (1.04 MB TIF) [file pone.0007650.s003.tif]

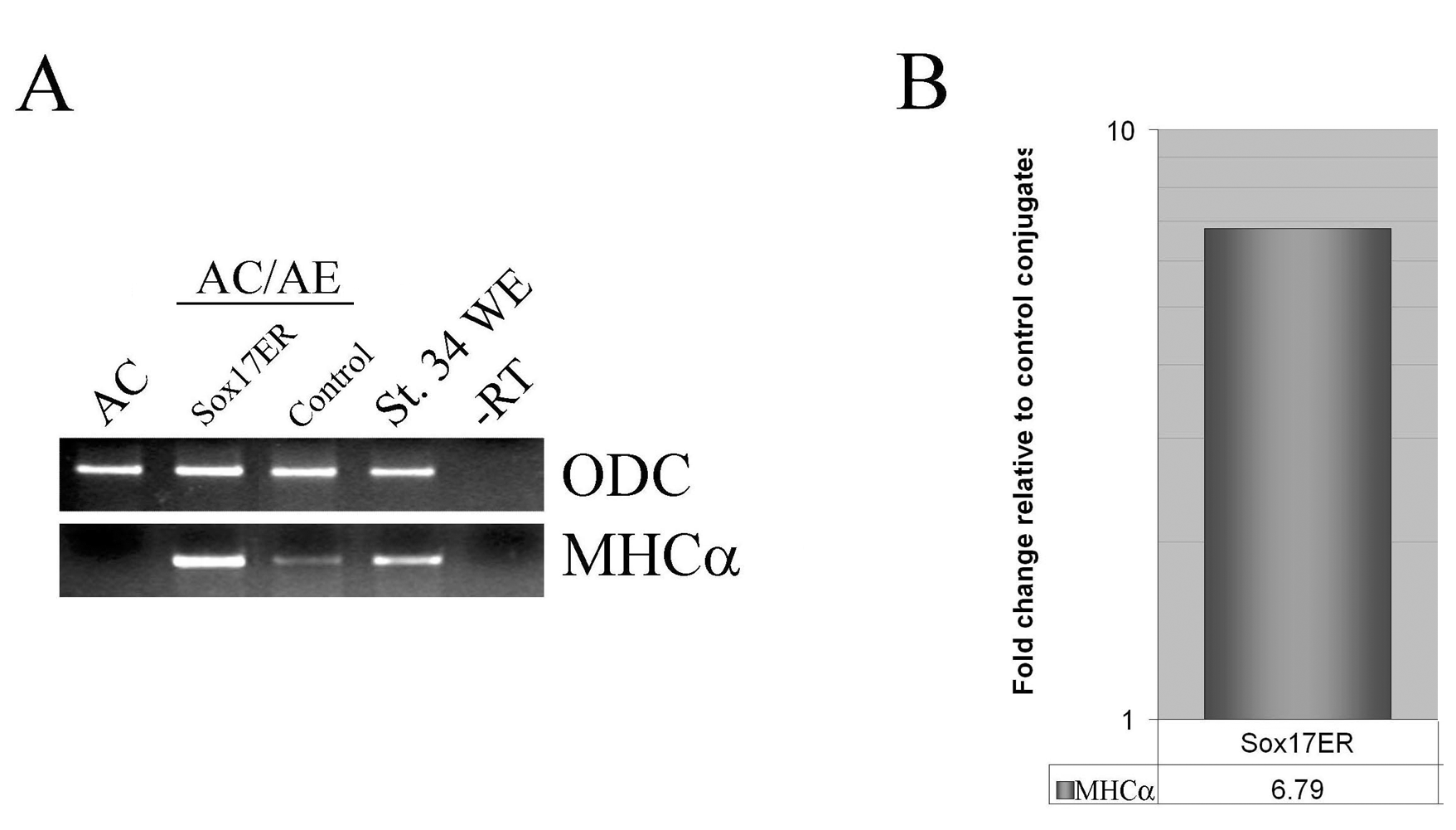

Supplement: Figure S4 — Sox17-dependent endoderm in responding tissue is not required for cardiogenesis in AC/AE model. (A) Animal caps from embryos injected with Sox17beta-Engrailed Repressor (Sox17ER) were conjugated with anterior endoderm and analyzed for MHCalpha expression when sibling control embryos reached st. 34. (B) Quantification of a gel in A, confirming that Sox17ER enhances cardiogenesis. (1.76 MB TIF) [file pone.0007650.s004.tif]

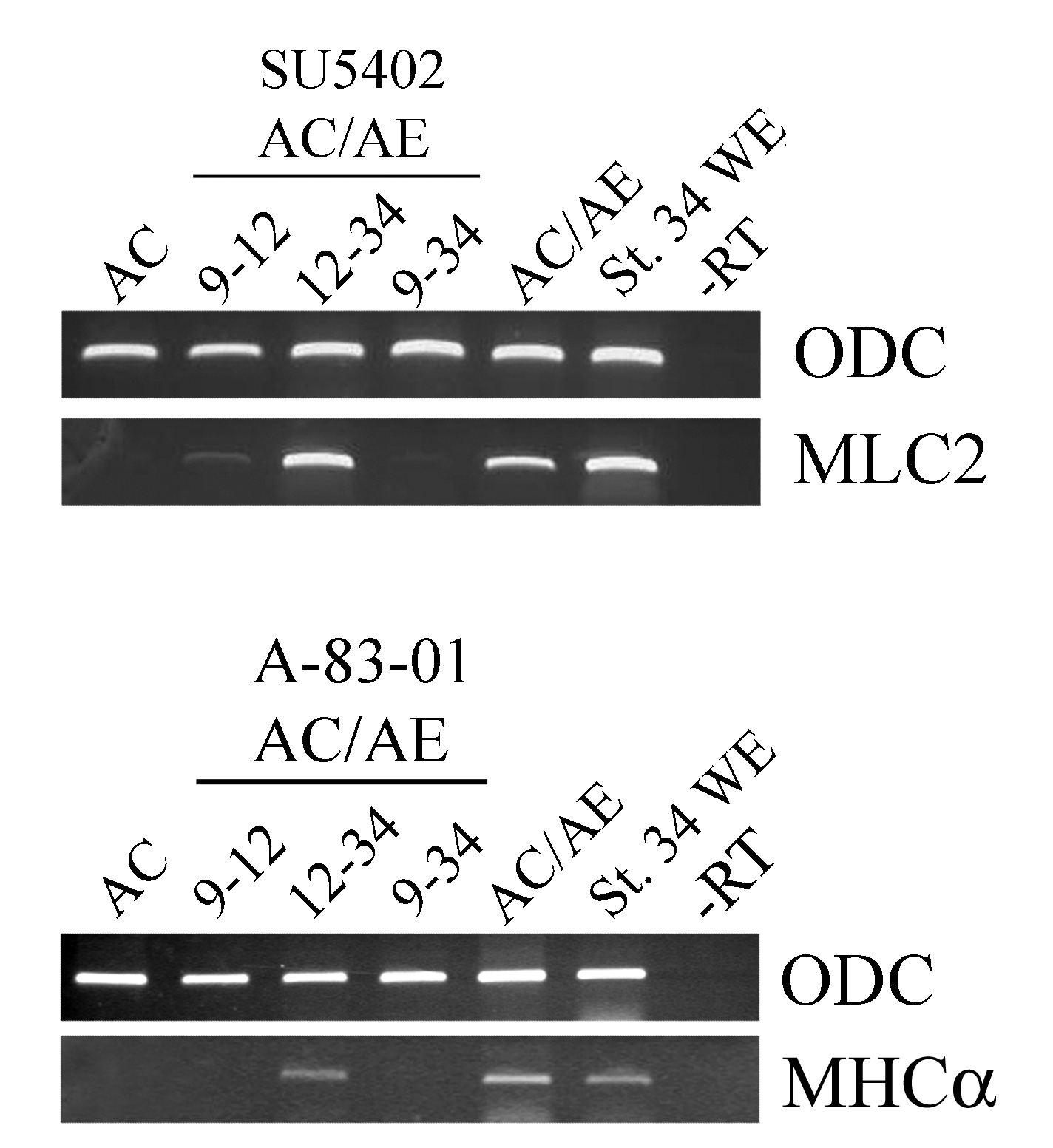

Supplement: Figure S5 — FGF and Nodal signalling are required from st. 9-st.12 (9–12), but not after st. 12 (12–34). (2.22 MB TIF) [file pone.0007650.s005.tif]

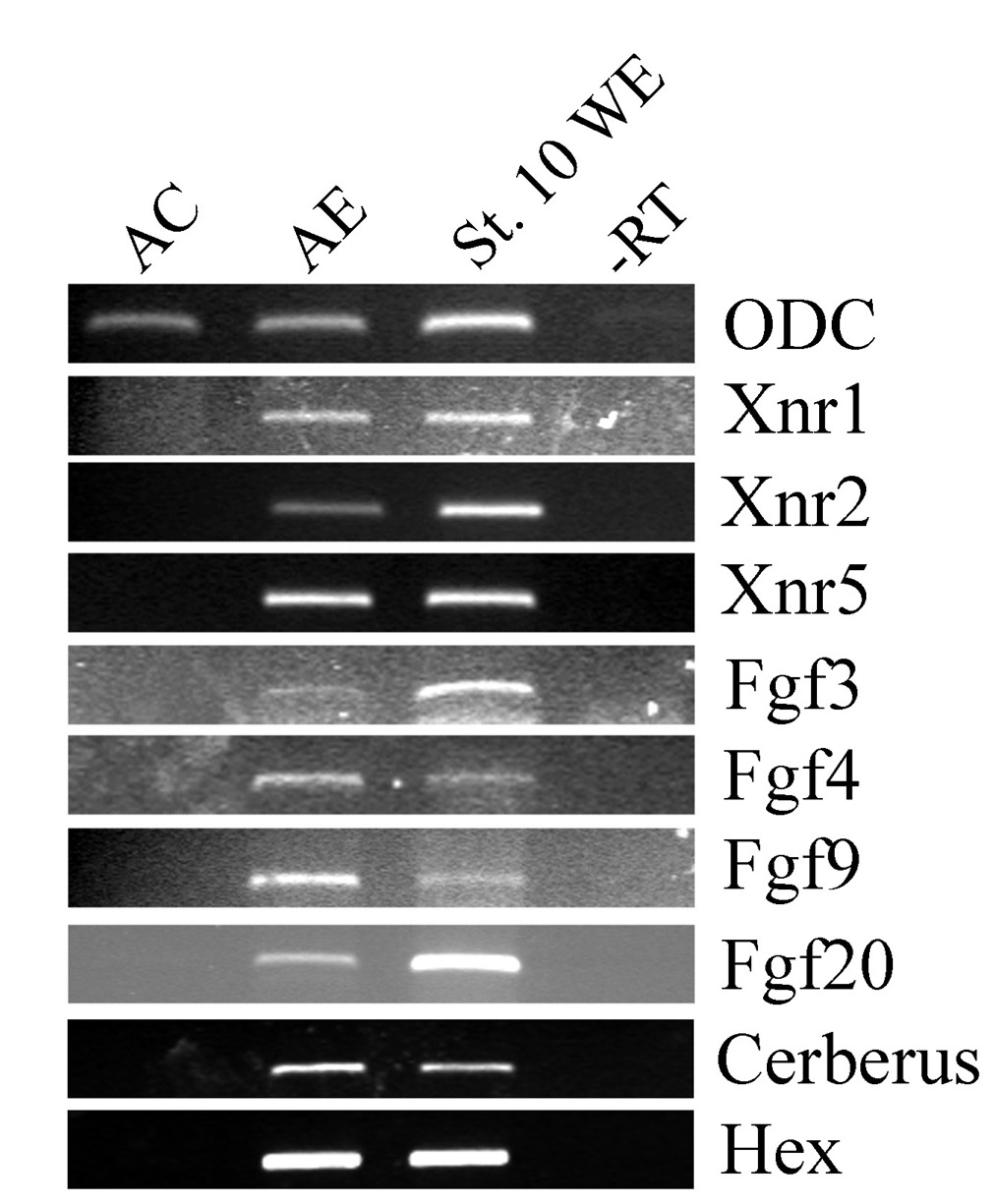

Supplement: Figure S6 — Anterior endoderm explants express several Nodal and FGF genes. Anterior endoderm explants were analyzed for expression of indicated genes immediately after excision. All genes tested are expressed in st. 10 anterior endoderm. Anterior character of endoderm explants was confirmed by expression of Cerberus and Hex. (1.25 MB TIF) [file pone.0007650.s006.tif]
