# Supplementary material for: Early Activation of FGF and Nodal Pathways Mediates Cardiac Specification Independently of Wnt/β-Catenin Signaling
Source: PLoS One. 2009 Oct 28;4(10):e7650. doi: 10.1371/journal.pone.0007650 (PMC2763344; doi:10.1371/journal.pone.0007650)
Supplement: Table S1 — Information about the primers used in the study. (0.13 MB DOC) [file pone.0007650.s007.doc]

| ***Primer*** | ***Sequence*** | ***Accession Number*** | ***Tm (°C)*** | ***Product Size (bp)*** | Cycle Number |
| --- | --- | --- | --- | --- | --- |
| *Cerberus (+)*  *Cerberus (-)* | 5’-gct tgc aaa acc ttg ccc tt-3’  5’-ctg atg gaa cag aga t-3’ | CR762343 | [1] | | |
| *Chodrin (+)*  *Chordin (-)* | 5’-ctt cgg aaa gac ccc aga gc-3’  5’-acg tgt acc agc tgc ctt cc-3’ | NM_001088309 | 58 | 300 | 32 |
| *cTnI (+)*  *cTnI (-)* | 5’-gag ctt cac gca aga att ga-3’  5’-gca tca acg ttc ttt ctc ca-3’ | BC088784 | 58 | 314 | 34 |
| *Endodermin (+)*  *Endodermin (-)* | 5’-CTC GAA GAG CCT CGA GA-3’  5’-ATT CCG GCT TTC CAG GTA GT-3’ | [L63543](http://www.ncbi.nlm.nih.gov/entrez/viewer.fcgi?db=nucleotide&id=1929448) | 55 | 398 | 34 |
| *Eomesodermin (+)*  *Eomesodermin (-)* | 5’-gtg ccc agg tct tcc tct gt-3’  5’-tag cgc ctt tgt tgt tgg tg-3’ | NM_001088341 | 58 | 345 | 30 |
| *FGF3 (+)*  *FGF3 (-)* | 5’-GTC ATT TGT TTC CAG ACT TC-3’  5’-TAT CTG TAG GTG GTA CTT AG-3’ | Z25539 | [2] | | |
| *FGF4 (+)*  *FGF4 (-)* | 5’-CCG CTT TCT TTC CAG AGA AAC GAC -3’  5’-GTC CGG TAA AAC CTG GAT ATG AA -3’ | X62594 | 54 | 177 | 36 |
| *FGF8 (+)*  *FGF8 (-)* | 5’-CTG GTG ACC GAC CAA CTA AG-3’  5’-ACC AGC CTT CGT ACT TGA CA-3’ | NM_001090435 | 54 | 328 | 36 |
| *FGF9 (+)*  *FGF9 (-)* | 5’-tat ttc ggt gtg cag gat gc-3’  5’-cag ctc ccc ttt ctc gtt ca-3’ | BC170125 | 58 | 348 | 34 |
| *FGF20 (+)*  *FGF20 (-)* | 5’-tgc tct tca acg acc cac tg-3’  5’-gtt ccc gaa aaa tgc act cg-3’ | NM_001090297 | 58 | 315 | 34 |
| *α-globin (+)*  *α-globin (-)* | 5’-ctg gcc atc cac ttc cat aa-3’  5’-tgt taa cac cgt cta acc tca gc-3’ | [X02796](http://www.ncbi.nlm.nih.gov/entrez/viewer.fcgi?db=nucleotide&id=64538) | 58 | 145 | 32 |
| *Goosecoid (+)*  *Goosecoid (-)* | 5’- GGA TTT TAT AAC CGG ACT GTG G-3’  5’- TGT AAG GGA GCA TCT GGT GAG -3’ | [M81481](http://www.ncbi.nlm.nih.gov/entrez/viewer.fcgi?db=nuccore&val=214185) | 58 | 250 | 34 |
| *Hex (+)*  *Hex (-)* | 5’-AGG CCA GTC AGC GAC TAC A-3’  5’-ATT TCC CTG TGG GTT CTC CT-3’ | NM_001085590 | 58 | 297 | 32 |
| *LURP-1 (+)*  *LURP-1 (-)* | 5’-ttt tgg tcg ttt tgg gga tg-3’  5’-gca gaa ttt tgg ggt ctt tgc-3’ | NM_001088645 | 58 | 301 | 30 |
| *Mesp-1 (+)*  *Mesp-1 (-)* | 5’-AAG AAG GCT AGC CGA AAT CC-3’  5’-CCA TCT GAG CCT GAA GCT GT-3’ | [DQ096961](http://www.ncbi.nlm.nih.gov/entrez/viewer.fcgi?db=nucleotide&id=70672202) | 58 | 371 | 36 |
| *MHCα (+)*  *MHCα (-)* | 5’-ACC AAG TAC GAG ACT GAC GC-3’  5’-CTC TGA CTT CAG CTG GTT GA-3’ | NM_001091601 | 58 | 597 | 32 |
| *MLC1 (+)*  *MLC1 (-)* | 5’-CGG ATC AAA CAG GAC GAT TC-3’  5’-GAA CCC CTG GTA GTG CAG AA-3’ | L38596 | 58 | 216 | 30 |
| *MLC2 (+) **  *MLC2 (-)* | 5’-TGT ATC GAC CAA AAC CGT GA-3’  5’-CTT CTG GGT CCG TTC CAT TA-3’ | Z33999 | 58 | 186 | 30 |
| *MSR (+)*  *MSR (-)* | 5’-ctc agg gaa tgg agt ggt ca-3’  5’-tgg caa cat tgc tcc aca atc c-3’ | X93045 | 58 | 245 | 32 |
| *Myf5 (+)*  *Myf5 (-)* | 5’-ACG AGC ATG TCA GAG CAC CT -3’  5’-ATC TCC ACC TTG GGC AGT CT -3’ | NM_001101779 | 56 | 214 | 34 |
| *MyoD (+)*  *MyoD (-)* | 5’-AGA GGA ACC CCA CCA TAA CG -3’  5’-TGA GGT GTA TCG CTT CAG GG -3’ | NM_001085897 | 56 | 202 | 32 |
| *Nkx2.5 (+)*  *Nkx2.5 (-)* | 5’-GAG CTA CAG TTG GGT GTG TGT GGT -3’  5’-GTG AAG CGA CTA GGT ATG TGT TCA-3’ | BC056048 | 58 | 250 | 34 |
| *N-tubulin (+)*  *N-tubulin (-)* | 5’-gca ttg atc cta cag gca gt-3’  5’-tgg gtc agt tga aaa cct tg-3’ | X15798 | 58 | 424 | 28 |
| *ODC (+) **  *ODC (-)* | 5’-GCC ATT GTG AAG ACT CTC TCC ATT-3’  5’-TTC GGG TGA TTC CTT GCC AC-3’ | NM_001086698 | 58 | 220 | 28 |
| *Scl (+)*  *Scl (-)* | 5’-gca atg tcc cta aag atg atg g-3’  5’-ctg cag tct cag ctc ctg ct-3’ | AF060151 | 58 | 236 | 34 |
| *Siamois (+)*  *Siamois (-)* | 5’-aag gaa ccc cac cag gat aa-3’  5’-ctg gta ctg gtg gct gga ga-3’ | Z48606 | 58 | 274 | 32 |
| *SmActin (+)*  *SmActin (-)* | 5’-cca att gaa cac ggc atc at-3’  5’-gca tga ggg aga gca tac cc-3’ | AY986490 | 58 | 314 | 30 |
| *Sox17α (+)*  *Sox17α (-)* | 5’-GAT GGT GGT TAC GCC AGC GA-3’  5’-TGC GGG GTC TGT ACT TGT AG-3’ | NM_001088162 | 58 | 377 | 34 |
| *Tbx5 (+)*  *Tbx5 (-)* | 5’-TCA GAA CCA CAA GAT CAC ACA G-3’  5’-GCT CAG CTG GCT CTT CAC TT-3’ | BC170344 | 58 | 354 | 34 |
| *Vent2 (+)*  *Vent2 (-)* | 5’-ACC TGC CAT GGA CTC TCT GA -3’  5’-ATG TCA ACA CAT GGC CCA AT-3’ | NM_001088138 | 56 | 267 | 32 |
| *XAG (+)*  *XAG (-)* | 5’-CTG ACT GTC CGA TCA GAC-3’  5’-GAG TTG CTT CTC TGG CAT-3’ | [NM_001086198](http://www.ncbi.nlm.nih.gov/entrez/viewer.fcgi?db=nucleotide&id=148237475) | [3] | | |
| *Xbra (+)*  *Xbra (-)* | 5’-ctg gga tgt tgc caa tga gt-3’  5’-gat gaa agc ctg gaa tgt gc-3’ | NM_001090578 | 58 | 282 | 32 |
| *XK81 (+)*  *XK81 (-)* | 5’-TCA AGA AGA ACC ACG AGG AG-3’  5’-CCG TTT CAG CTC AGT GAT TT -3’ | M11940 | 56 | 263 | 32 |
| *Xnr1 (+)*  *Xnr1 (-)* | 5’-TGG CCA GAT AGA GTA GAG -3’  5’-TCC AAC GGT TCT CAC TTT -3’ | [NM_001085796](http://www.ncbi.nlm.nih.gov/entrez/viewer.fcgi?db=nucleotide&id=147905879) | 52 | 250 | 36 |
| *Xnr2 (+)*  *Xnr2 (-)* | 5’-tgg gca atc gat gga cat ta -3’  5’-tga cat gtg gct tgg ctc tc -3’ | NM_001087967 | 54 | 321 | 34 |
| *Xnr3 (+)*  *Xnr3 (-)* | 5’-gtt tcc cca att cat gat gc-3’  5’-agc tca gcc aac ttc agc ctc-3’ | BC169689 | 58 | 206 | 30 |
| *Xnr5 (+)*  *Xnr5 (-)* | 5’-ggg atg ccc act ctt ctt ca-3’  5’-ctc cgc cag cct taa ctc ac-3’ | AB038133 | 54 | 318 | 34 |
| *Xpo (+)*  *Xpo (-)* | 5’-GCT GAT TAC CAT TCA TGT GCA G -3’  5’-TCA CCT CTT GTT CTC TGA GCC -3’ | X58487 | 56 | 394 | 34 |

* these primers were also used for qPCR

1. Zamparini, A.L., et al., *Hex acts with beta-catenin to regulate anteroposterior patterning via a Groucho-related co-repressor and Nodal.* Development, 2006. **133**(18): p. 3709-22.

2. Kofron, M., et al., *Mesoderm induction in Xenopus is a zygotic event regulated by maternal VegT via TGFbeta growth factors.* Development, 1999. **126**(24): p. 5759-70.

3. Walters, Z.S., K.E. Haworth, and B.V. Latinkic, *NKCC1 (SLC12a2) induces a secondary axis in Xenopus laevis embryos independently of its co-transporter function.* J Physiol, 2009. **587**(Pt 3): p. 521-9.
